# Practices and Barriers towards Physical Assessment among Nurses Working in Intensive Care Units: Multicenter Cross-Sectional Study

**DOI:** 10.1155/2021/5524676

**Published:** 2021-07-14

**Authors:** Bikis Liyew, Ambaye Dejen Tilahun, Tilahun Kassew

**Affiliations:** ^1^Department of Emergency and Critical Care Nursing, School of Nursing, College of Medicine and Health Sciences, University of Gondar, Gondar, Ethiopia; ^2^Department of Psychiatry, School of Medicine, College of Medicine and Health Sciences, University of Gondar, Gondar, Ethiopia

## Abstract

**Background:**

In the intensive care units, patients need special consideration and monitor frequently with appropriate physical assessment skills. Nurses working in the intensive care units play a fundamental role in detecting patients at risk of deterioration through ongoing assessment and action in response to changing health status. Most of the nursing activities were poorly assessed in low-income countries including Ethiopia. Therefore, this study was aimed to assess the nurses' practice and barriers to physical assessment among critically ill patients in Northwest Ethiopia.

**Methods:**

An institution-based multicenter cross-sectional study was conducted at Amhara regional state referral hospitals from March to September 2019. A total of 299 nurses working in the intensive care units were recruited through the convenience sampling method. A 30-item physical assessment practice and 36-item barriers to nurses' use of the physical assessment scale inventory were used. The linear regression analysis model was fitted, and the adjusted unstandardized beta (*β*) coefficient with a 95% confidence interval was used. A *p* value < 0.05 was considered statistically significant.

**Results:**

The mean score of the nurses' practice towards physical assessment among critically ill patients was 101.26 ± 24.99. Greater perceived reliance on others and technology (*β* = −0.78, 95% CI (-1.07, -0.48)), ward culture (*β* = −0.48, 95% CI (-0.85, -0.11)), specialty area (*β* = −1.46, 95% CI (-2.01, -0.90)), lack of nursing role model (*β* = −0.54, 95% CI (-1.06, -0.02)), being unmarried (*β* = −6.10, 95% CI (1.75, 10.46)), taken training (*β* = 11.53, 95% CI (6.34, 16.72)), and knowledge score (*β* = 2.81, 95% CI (2.00, 3.63)) were the factors significantly associated with the nurses' practice score towards physical assessment. Reliance on others and technology towards physical assessment practice was the most important barrier followed by ward culture and specialty area.

**Conclusion:**

Nurses working in the intensive care units had a good practice towards physical assessment among critically ill patients. Hence, to increase the practice towards physical assessment in intensive care settings, especially for married nurses, experienced critical care nurses, and specialist professionals, practice support training, modifying ward environment, and educational support care are recommended.

## 1. Background

Physical assessment is an organized systemic process of collecting objective and subjective data based upon a health history and head-to-toe or general body systems examination by using the skills of inspection, auscultation, percussion, and palpation [1–3]. European countries included physical assessment skills into their nursing practice as a health assessment component [4]. A systematic way of physical assessment ensures better patient outcomes and improves patient quality of life, and helps to obtain baseline physical data, establish nursing diagnoses and action plan for patient care, and evaluate the appropriateness of the nursing interventions and care outcomes [3, 5, 6]. It also helps the nurses' to summarize, interpret, and document the clinical findings and recognition of abnormality or identification of a differential diagnosis in order to make clinical decision [7, 8]. Therefore, performing a correct physical assessment is an essential skill in which all healthcare professionals must possess [2, 9, 10]. Nurses are integral members of a multidisciplinary healthcare team in an intensive care unit, and they often have the responsibility and privilege of performing a focused physical assessment for each of their patients to provide an important opportunity to evaluate and formulate a plan of care [11, 12]. It is a core competency within the scope of nursing practice [1, 7, 13–15].

Nurses must have acquired new skills to allow them to diagnose and treat patients [16, 17]. A deteriorating patient moves from one clinical state, increasing the risk of morbidity and death [18]. Even though physical assessment skills are key components of nursing practice, failure to recognize hospitalized patients at risk of clinical deterioration is growing evidence due to inadequate physical assessment practice of nurses [19]. To know about a patient's health condition, and to check the quality of nursing care, each hospitalized patient should receive a day-to-day physical assessment by a trained nurse [20, 21]. Some studies revealed that nurses do not perform physical assessment practices [1, 13, 22], or that they perform only a small number of practices [13, 22]. Physical assessment skills predominantly performed by nurses regularly for critically ill patients were vital signs [18, 23–25]. Physical assessment competency performance requires nursing skills for better decision-making and improved clinical reasoning and performance for critically ill patients [24, 26]. Adequate practice skill and awareness about physical assessments plays a vital role in early diagnosis, appropriate management, and alleviation of adverse consequences resulting from critical problems [27, 28]. Critical care nurses need to possess advanced skills, competencies, and capabilities to care for critically ill patients [29].

The study conducted in Australia by Birks et al. reported that practice was influenced by the lack of time available to complete assessments, areas of clinical practice, or specialty and the presence or absence of other healthcare workers, such as medical and allied health staff [13]. Previous works of literature reported that lack of training; failure of the organization; lack of supervision; absence of a uniform physical assessment form; lack of time, support, and encouragement [30, 31]; lack of time and heavy workload [32]; lack of knowledge, lack of confidence in skills, and lack of work experience [33, 34]; and reliance on others and technology, lack of time and interruptions, ward culture, lack of nursing role models, lack of influence on patient care, and specialty areas [35] were some of the barriers faced by nurses. In addition, different literature reviews showed that ICU experience, age, sex, and education level, the attitude of individuals affects the practice of ICU nurses [18, 33, 36, 37]. Physical assessment is necessary to improve health care quality and to develop patient care plans [38]. The ability to perform a physical assessment is not only a crucial component of the nursing process but also a basic skill all nurses must have [10]. Conducting physical examination in the modern ICU is challenged due to repositioning patients, clinical instability, the presence of bulky dressings, lines and tubes, electrocardiograph (ECG) monitors, and high levels of ambient ICU noise represent common barriers [39, 40]. Due to the increasing complexities are seen within the world of healthcare today, healthcare professionals that have proper physical assessment skills and abilities are more important than ever. A physical assessment is the responsibility of both nurses and physicians alike physical assessments also help healthcare providers in identifying changes in a patient's condition and intervening quickly and appropriately. In Ethiopia, physical assessment is an integral part of the nursing curriculum and training in undergraduate studies, advanced courses, and postgraduate studies in critical care nursing. However, despite the theoretical and practical training that nurses receive during their studies, it is not outstandingly evident in the ICU. Even though ICU nurses' physical assessment practices have an impact on the quality of care for critical care patients, no study shows the practice of nurses working in the ICU regarding physical assessment and its influencing factors [41, 42]. Therefore, identifying nurses' practices regarding physical assessment skills is crucial for improving the patient's plan of care to the developed nursing process, diagnosis, and interventions and quality of life. Therefore, the result of this study will help policymakers and stakeholders to develop and carry out evidence-based actions for helping nurses to improve their practice towards physical assessment. Therefore, this study was aimed to investigate nurses' physical assessment practices and what barriers they encounter when attempting to perform these skills in the Amhara region, Northwest Ethiopia.

### 1.1. Conceptual and Theoretical Framework of Physical Assessment

**Figure 1:**
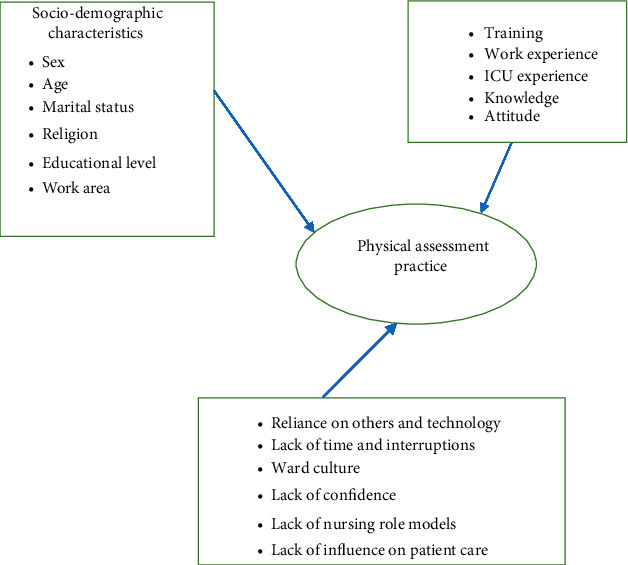
Physical assessment conceptual framework.

A physical assessment conceptual framework can be used by critical care nurses as a guide to the process of conducting a physical assessment. It includes focused and comprehensive health history through inspection, palpation, percussion, and auscultation to interpret, recognize abnormality, and differentiate the diagnosis of critically ill patients to make clinical decision. Barriers to physical assessment practice contain seven subscales such as reliance on others and technology, lack of time and interruptions, ward culture, lack of confidence, lack of nursing role models, lack of influence on patient care, and specialty area [8, 35, 43–47] (Figure 1).

## 2. Methods

### 2.1. Study Setting

A multicentered, institution-based cross-sectional study was conducted to assess the practice and barriers about physical assessment among nurses working in the intensive care units at Amhara regional state referral hospitals, Northwest Ethiopia from March to September 2019. Amhara region is the second-largest region in Ethiopia, with vast climatic, geographical, and cultural diversity. According to the central statistical agency of Ethiopia, health and health-related indicators reported by the federal ministry of health (FMoH) 2015/16; in the Amhara region, there are five referral hospitals; Felege Hiwot, Dessie, Debre Markos, Debre Berhan, and University of Gondar referral hospitals in which intensive care for neonates, pediatric, and adult patients are provided. These hospitals provide specialized outpatient and inpatient services in different departments including emergency, surgical, internal medicine, gynecology and obstetrics, psychiatry, intensive care units (neonatal, pediatrics, and adult surgical and medical), ophthalmology, pediatrics, and oncology. Each hospital provides services for an estimated five million population. The hospitals have their own organized neonatal, pediatric, and adult intensive care units [48].

### 2.2. Study Population

A total of 299 nurses working in the intensive care units were recruited using convenience sampling techniques with a response rate of 95.6%. Since the study population was minimal for the adequacy of sample size, all nurses working in adult, pediatric, and neonatal ICUs of Felege Hiwot, Dessie, Debre Markos, Debre Berhan, and University of Gondar referral hospitals were invited to participate in the study. Nurses working in the intensive care units with six months' and more work experience were included, while those nurses with annual leave and sick leave during data collection and nursing personnel not involved in the direct management of the patients (e.g., nursing managers and tutorial staff) were excluded [48].

### 2.3. Data Collection Tool

Data were collected using structured, self-administered questionnaires. The questionnaires were adopted and modified from previous kinds of literature [13, 22, 24, 35, 48, 49]. The questionnaire consisted of four sections such as sociodemographic data, physical assessment practice questionnaire, barriers to nurses' use of physical assessment scale inventory, and knowledge and attitude questionnaire.

#### 2.3.1. Physical Assessment Practice Questionnaire

This tool was initially developed by Giddens [22] and, then, improved by Briks et al. [13] again which was modified and validated by Cicolini et al. [24] with Cronbach's alpha value of 0.94 and total item correlation ranged between 0.38 and 0.72. The original tool developed by Giddens had 126 items for assessing physical examination skills are most commonly performed by practicing nurses. Cicolini et al. modified and reduced the tool to 30 items routinely taught and performed according to the Italian bachelor degree requirements and included them in the final questionnaire. The researchers in this study used 30 items of the physical assessment skill inventory validated by Cicolini et al. The responses to each question were a six-point Likert scale such as 0 = I do not know how to do, then not to do this technique, 1 = I know how to do this technique, but it is not part of my clinical practice, 2 = I perform this technique rarely (a few times), 3 = I perform this technique occasionally (a few times per year), 4 = I perform this technique frequently in my clinical practice (every 2–5 times I work), and 5 = I perform this technique regularly in my clinical practice (every time I work). The possible ranges of the total score of the tool were 0-150. Individuals with the total score approach to “0” were considered as very poor practice and the scoring approach to “150” was considered as the best possible practice.

#### 2.3.2. Barriers to Nurses' Use of the Physical Assessment Scale Inventory

This tool was initially developed by Douglas et al. (51), which was used to assess the nurse's barrier towards physical assessment with internal reliability ranging from 0.70 to 0.86. The nurse's barrier towards the physical assessment questionnaire with a 5-point Likert-type scale from 1 = strongly disagree to 5 = strongly agree. This contains seven subscales with a total of 36 items factored into seven subscales: (i) reliance on others and technology (9 questions), (ii) lack of time and interruption (5 questions), (iii) ward culture (5 questions), (iv) lack of confidence (4 questions), (v) lack of nursing role models (4 questions), (vi) lack of influence on patient care (4 questions), and (vii) specialty area (5 questions). The negative Likert scale questions were reversely coded. The possible ranges of the total score of barriers towards physical assessment were converted into a sum of each subscale.

#### 2.3.3. Knowledge and Attitude Questionnaire

The knowledge and attitude questionnaire was adopted by reviewing previous kinds of literature [48] and developed by a panel of emergency and critical care professionals, by reviewing previous gray literature [49] based on the standard of tool development [50], and by evaluating its content and face validity, clarity, and discrimination of items. The knowledge questionnaires were consisted of 15 multiple choice questionnaires. The possible range of the total score of knowledge on physical assessment was 0-15, where the score closer to 0 indicate poor knowledge and closer to 15 indicates the best possible knowledge. On the other hand, the attitude questionnaires consisted of 10 items. From those, 5 of the items were worded positively and 5 were phrased negatively. The negative Likert scale questions were reversely coded. The possible range of the total score of attitude on physical assessment was 10 to 50, where the score closer to 10 indicates poor attitude and closer to 50 indicates the best possible attitude.

### 2.4. Data Collection Procedures and Quality Control

Data were collected by five nurses who were the head of each hospital's intensive care unit who distributed the questionnaires to the respondents for getting their willingness and collected the filled data. The questionnaire format was filled in their clinical area by the respondent nurses in the presence of the data collectors. The questionnaire was designed in English and was translated to Amharic, the official language of Ethiopia, and back to English, forward and backward translation for its consistency. The pilot study was done among 15 nurses working in ICU conducted at Saint Paulo's referral hospital in Addis Ababa. The face and content validity of the tool was examined by the researchers and critical care professionals. The Cronbach's alpha coefficient for the practice questionnaires was 0.951 for assessing the internal consistency and reliability of the tool. Alpha coefficients were computed for each subscale of barrier and the total barrier scale of physical assessment. These values were reliance on others and technology (0.646), lack of confidence (0.657), lack of nursing role model (0.652), lack of time and interruption (0.551), ward culture (0.673), and specialty area (0.587) for each subscale. The overall barriers to the physical assessment scale were 0.825. Cronbach's alpha coefficient of the knowledge and attitude part of this study was 0.635 and 0.743, respectively.

### 2.5. Data Processing and Analysis

The collected data were checked for completeness and consistency and entered into epi-info version 7.2 and exported to STATA version 14 software for analysis. Both descriptive and analytical statistical procedures were used to summarize the distribution of variables. The assumption test was checked before conducting the regression analysis. Simple linear regression analysis was performed to test the correlation between practice towards physical assessment and each independent variable. All explanatory variables from a simple linear regression model were fitted into the multiple linear regression model, and finally, the variables which had been independent association with practice towards physical assessment were expressed as adjusted unstandardized *β* coefficient by 95% confidence level. A *p* value of < 0.05 was considered as statistically significant for all analyses, and the model fitness test (adjusted *R*^2^) was also checked.

## 3. Results

### 3.1. Sociodemographic and Work-Related Characteristics of Study Participants

Two hundred ninety-nine nurses were involved in this study with a response rate of 96.5%. More than half of the study participants were female 162 (54.2%). The mean age of the participants was 31.9 ± 3.81 years. Two hundred six (78.9%) of the study respondents were married, and the majority of the participants 263 (88.0%) were orthodox Christian. Two hundred forty-nine (83.3%) of the respondents were qualified for a Bachelor of Science degree. The mean total years of work experience and years of experience working in ICU were 5.7 ± 2.54 and 1.83 ± 0.798, respectively. The average monthly income was 5748.64 ± 1698.420 Ethiopian birr per month. The knowledge and attitude mean scores of nurses working in intensive care units were good (9.93 ± 2.99 and 36.85 ± 6.21, respectively) (Table 1).

### 3.2. Practice towards Physical Assessment among Nurses Working in ICU

The mean score of practice towards physical assessment among nurses was 101.26 ± 24.99 with 95% CI (98.23, 103.98). This study indicated that nurses working in the intensive care units had a good practice towards physical assessment among critically ill patients. The proportion of nurses who scored above the mean was 153 (51.2) with 95% CI (45.8, 56.9) and below the mean 146 (48.8) with 95% CI (43.1, 54.2). The minimum and maximum scores of practice towards physical assessment among nurses were 25 and 147, respectively. In this study, 88 (29.4%) of the respondents performed regularly the procedure of inspecting the pupil with equal size, shape, and relative to react with light, and 87(19.10%) of the participants responded that they regularly inspect skin lesions and wounds. Regarding auscultation, 101 (33.8%) and 108 (36.1%) of the nurses auscultate lung sound and heart sound, respectively. Regarding routine nursing activities, nasogastric tube and patient monitoring machines, 117 (39.1%), measuring oxygen saturation, 127 (42.5%), blood pressure 144 (48.2%), and body temperature 152 (50.8%) of the procedures were regularly performed by nurses working in ICU. On the other hand, extremities for tenderness 25 (8.4%), abdomen for tenderness and distension 27 (9.0%), hearing on the basis of conversation 37 (12.4%), and speech pattern 51 (17.0%) of the procedures were not known and did not perform by nurses for critically ill patients (Table 2).

### 3.3. Barriers towards Physical Assessment among Nurses Working in Intensive Care Units

The seven subscales' means were ranging from 12.18 (±4.180) (lack of time and interruptions) to 29.27 (±7.48) (reliance on others and technology). The most important barrier was “the information collected using physical assessment skills is used to make treatment decisions” (mean = 3.86, SD = 3.28, 72.6%, *n* = 217) from the lack of influence on the patient care subscale. Reliance on others and technology (mean ± SD = 29.27 (±7.48)) towards physical assessment practice was the most important barrier followed by ward culture (mean ± SD = 13.84 (±6.07)) and specialty area (mean ± SD = 13.02 (±4.37)) (Table 3).

### 3.4. Factors Associated with Practice towards Physical Assessment among Nurses Working in Intensive Care Units

Results of multiple linear regressions showed that reliance on others and technology, ward culture, lack of nursing role model, specialty, area, being unmarried, taking training, and knowledge of nurses were factors significantly associated with the total score of physical assessment practice of nurses. Regarding predictors, as the total score of reliance on others and technology (*β* = −0.78, 95% CI (-1.07, -0.48)) increased by a unit, nurses' practice towards physical assessment was decreased by 0.78 units. We would expect nurses' practice towards physical assessment to decrease by 0.48 units as the total score of ward culture (*β* = −0.48, 95% CI (-0.85, -0.11)) was increased by a unit. Lack of nursing role models (*β* = −0.54, 95% CI (-1.06, -0.02)) were to increase by one unit, and we would expect practice towards physical assessment to decrease by 0.541 units (Table 4).

## 4. Discussion

This is the first study done in Ethiopia about the practice of nurses towards physical assessment in intensive care settings. Research is needed to measure the practice and its barriers which predict the nurses' actual use of physical assessment. Greater attention is needed on the practice and the barriers of nurses' work practices because of poor practice resulting in a failure to recognize patients at risk of clinical deterioration [19]. Overall, the findings provide evidence for the magnitude and factors influencing nursing assessment practice in intensive care settings.

The mean score of practice towards physical assessment among nurses was 101.26 ± 24.99 (95% CI (98.23, 103.98)). However, it was difficult to extract raw data for comparison with our study due to the nature of categorizing and reporting, and there is no similar study done with the same regression analysis and description. In the current study, the proportion of nurses who scored above the mean was 153 (51.2) with 95% CI (45.8, 56.9) and below the mean 146 (48.8) with 95% CI (43.1, 54.2). This finding is higher than the study conducted in Australia, 70% of skills were not performed across the clinical settings. Of these, 42% of the skills were learned, but not practiced [35]. The possible reason might be the difference in the approaches of categorizing the total score result and analysis techniques. In our study, the approaches of categorization were using the mean (below and above) and described the mean and standard deviation. Therefore, it is expected to have a higher proportion than the previous study.

Findings in this study revealed that more than half 152(50.8%) of nurses reported that measuring body temperature must be performed regularly. Inspect overall skin integrity and color and inspect and palpate extremities for edema were the procedures known by all respondents on how to do the practice even if 11 (3.7%) and 18 (6.0%) of the respondents said that not our clinical practice task, respectively. Fifty-one (17.0%) of the participants do not know how to do evaluating speech patterns. Whereas, 65 (21.7%) of participants performed the procedure regularly. The primacy of routine nursing activities (temperature, blood pressure, breathing effort, oxygen saturation) in the intensive care units should be skill performed regularly to prevent the deterioration of the patient and serious adverse events [18, 19].

The results of the current study also investigated that practices towards physical assessment of unmarried nurses' were increased by 6.10 unit scores as compared to married nurses. This result is agreed with different studies [51, 52]. The possible reason was unmarried staff nurses might have adequate time and much attention to their work. This reduces nurses' work overload in the care of critically ill patients that might help them to have a good practice. However, married staffs have tensioned by extra daily activities related to family and child care. Being married is correlated with burnout, and this affects the practice of nurses towards physical assessment [51, 53].

The result of this study showed that nurses had taken a training related to physical assessment; the practice towards physical assessment increased by 11.53 units score as compared to those not having taken the training. This study is supported by previous studies [54–56]. Taken training towards physical assessment increases when opportunities for practice are provided and in ICUs setup know a day the development of advanced monitoring device's needs daily updated manual training and practice. This increases nurses' knowledge and practice, as evidenced by our study, 115 (38.5%) of the participants had taken training towards physical assessment. Professional training should be viewed as a continuum, which begins with basic training and should continue throughout professional life [57].

In this study, as the knowledge score increased by a unit, nurses' practice towards physical assessment increased by 2.81 units. This finding was consistent with the previous studies [15, 58]. If nurses working in critical care units have a lack of knowledge and confidence in their ability to assess critically ill patients, their attendants and supervisors reported frequently as a reason why nurses are not implementing physical assessment skills into their health assessment. This enforces them to read and develop their competencies. Hence, nurses working in ICUs may be thought that a critically ill patient needs knowledgeable and evidence-based practice. Supervising nursing professionals has a value in promoting safe and effective patient care and enabling the development of professional skills [59]. This makes that having good knowledge increases their practice towards physical assessment skills. Arguably, nurse educators have to reevaluate physical assessment course content and focus on “what nurses need to know to practice nursing” [1].

The results of the study showed that greater perceived reliance on others and technology was associated with skill utilization of physical assessment. This finding was consistent with other studies [1, 35]. There is a dramatic increment in the production and utilization of different vital sign measurement devices in the critical care setting. The possible justification might be due to the nurses' inclination to integrate physical assessment using advanced technologies and vital sign monitoring devices during the face of tension, and this affects the nurse's practice to utilize advanced physical assessment skills systematically. The other reason possible reason may be the medical model drives the culture of physical assessment techniques that are designed for diagnostic purposes. However, diagnosing is within the agreement of both disciplines for patient assessment and diagnosis [26]. Therefore, nurses working in ICUs must be ready for how to use technology and could improve the nursing physical assessment practices in intensive care units.

Lack of a nursing role model subscale was associated with the nurses' practice towards physical assessment. This result is in agreement with other studies held in the Arab Peninsula [47]. Lack of experienced consultant nursing professionals in physical assessment practice contributed to the low performance in critical care and inadequate patient monitoring services. This might be to reduce the quality of care in the ICUs. Decision-making, self-efficacy in practice, and a better understanding of frontline caregiving in a critical care setup were undermined and disrupted work performance in physical assessment skills without knowledge sharing [13, 60]. This study showed that ward culture and specialty area barrier subscales were associated with the practice towards physical assessment among nurses working in the ICUs. This finding is supported by previous studies [25, 35, 47]. This could disrupt their work performance in physical assessment practice, leading to physical inadequacies, and increase diagnostic errors. Those barriers could be manifested in the uncooperative ward culture, which could hinder the professional development of nursing practice [13]. Arguably, health care institutions need to change their culture [31]. In our context, comprehensive nurses are professionals working in the ICUs as critical care nurses which deviate the standard of care about a specific specialty. This may affect the patients' care and support quality. In this study, training was a highly significant factor to use physical assessment skill practice. This finding was consistent with other studies conducted in Japan [61]. Therefore, to improve caregiving quality through systematic and advanced physical assessment skills, having a well-trained critical care nurse is crucial. Indeed, this reveals that training for nursing staff concerning the physical assessment of intensive care patients is crucial.

## 5. Conclusion

Based on the results of this study, nurses working in the intensive care units had a good practice towards physical assessment among critically ill patients. Reliance on others and technology, ward culture, specialty area, being unmarried, taking training, knowledge score, and lack of nursing role model were barriers for nurses' good practice towards physical assessment in ICUs' settings. Hence, experienced critical care nurses, specialized professionals, practice support training, modifying ward environment, and educational support are recommended especially for married nurses to increase practice towards physical assessment skills in ICUs.

### 5.1. Strength of the Study

This study was probably the first research related to the practice of physical assessment among nurses in Amhara regional state referral hospitals and in the country Ethiopia. It will be helpful as baseline information for other researchers.

### 5.2. Limitations of the Study

Since this study had used a self-administered questionnaire rather than observational checklists to measure the nurse's practice regarding physical assessment, the following limitations were inherent: avoidance of using extreme response categories by participants—central tendency bias, agreeing with statements as presented—acquiescence bias and participants attempt to portray themselves or their organization in a more favorable way social desirability bias. Therefore, the result may not reflect the actual nursing practice regarding physical assessment. The study design was cross-sectional, and therefore, it cannot establish cause and effect relationships. Since this was a quantitative study, it may not explore all associated factors, and it is advisable to use both quantitative and qualitative methods as well. Study results are not generalizable due to a convenience sampling technique. The study used self-reported surveys to collect data, and the study's surveys did not contain an area for narrative responses.

## Figures and Tables

**Table 1:** Sociodemographic and work-related characteristics of participants at Amhara regional state referral hospitals, Northwest, Ethiopia, 2019 (*n* − = 299).

Variables	Categories	Frequency (*N* = 299)	Percentage (%)
Sex	Male	137	45.8
	Female	162	54.2
Marital status	Married	236	78.9
	Unmarried	63	21.1
Religion	Orthodox	263	88.0
	Muslim	21	7.0
	Protestant	15	5.0
Educational level	Diploma	8	2.7
	Degree	249	83.3
	Master	42	14.0
Work area currently employed	Adult ICU	190	63.5
	Pediatric ICU	22	7.4
	Neonatal ICU	87	29.1
Training	Yes	115	38.5
	No	184	61.5
	Mean (standard deviation)		
Age	31.9 (3.81)		
Monthly income	5748.64 (1698.420)		
Total years of experiences	5.7 (2.54)		
Work experiences at ICU	1.83 (0.798)		

Note: ∗ICU: intensive care unit.

**Table 2:** Practice of physical assessment and skill performance of study participants at Amhara regional state referral hospitals, Northwest Ethiopia, 2019 (*n* = 299).

Practice item	Responses *n* (%)					
	I do not know how to do this technique	I know how to do but not my clinical practice	I perform this technique rarely	I perform this technique occasionally	I perform this technique frequently	I perform this technique regularly
Inspect external eyes and hair growth	31 (10.4)	68 (22.7)	57 (19.1)	21 (7.0)	66 (22.1)	56 (18.7)
Pupil equal size, shape, and relative to react light	4 (1.3)	35 (11.7)	68 (22.7)	55 (18.4)	49 (16.4)	88 (29.4)
Inspect oral cavity	7 (2.3)	32 (10.7)	60 (20.1)	76 (25.4)	73 (24.4)	51 (17.1)
Inspect chest shape	11 (3.7)	7 (2.3)	68 (22.7)	85 (28.4)	63 (21.1)	65 (21.7)
Inspect abdominal area	7 (2.3)	20 (6.7)	64 (21.4)	58 (19.4)	78 (26.1)	72 (24)
Inspect genital area	14 (4.7)	42 (14.0)	58 (19.4)	71 (23.7)	61 (20.4)	53 (17.7)
Inspect extremities	7 (2.3)	21 (7.0)	36 (12.0)	96 (32.1)	73 (24.4)	66 (22.1)
Inspect muscle and limps sizes	4 (1.3)	25 (8.4)	47 (15.7)	64 (21.4)	82 (27.4)	77 (25.8)
Inspect the spine	18 (6.0)	40 (13.4)	42 (14.0)	82 (27.4)	52 (17.4)	65 (21.7)
Inspect skin lesion and wound	14 (4.7)	21 (7.0)	37 (12.4)	69 (23.1)	71 (23.7	87 (29.10)
Inspect overall skin integrity and color	0 (0)	11 (3.7)	36 (12.0)	109 (36.5)	64 (21.4)	79 (26.4)
Inspect and palpate extremities for edema	0 (0)	18 (6.0)	48 (16.1)	63 ((21.1)	71 (23.7)	99 (33.1)
Observe the range of motion of joints	15 (5.0)	22 (7.4)	62 (20.7)	52 (17.4)	98 (32.8)	50 (16.7)
Evaluate the face for movements and sensation	15 (5.0)	11 (3.7)	64 (21.40	44 (14.7)	100 (33.4)	65 (21.7)
Evaluate breathing effort	29 (9.7)	4 (1.3)	64 (21.4)	63 (21.1)	90 (30.1)	49 (16.4)
Assessment of mental status and GCS	15 (5.0)	21 (7.0)	106 (35.50	43 (14.4)	64 (21.4)	50 (16.7)
Palpate and inspect capillary refill	21 (7.0)	14 (4.7)	36 (12.0)	67 (22.4)	73 (24.4)	88 (29.4)
Palpate distal pulse for circulation	18 (6.0)	22 (7.4)	7 (2.3)	71 (23.7)	101 (33.8)	80 (26.8)
Auscultate lung sound	23 (7.7)	26 (8.7)	36 (12.0)	57 (19.1)	56 (18.7)	101 (33.8)
Auscultate for heart sound	23 (7.7)	37 (12.4)	39 (13.0)	50 (16.7)	42 (14.0)	108 (36.1)
Auscultate abdomen for bowel sounds	23 (7.7)	40 (13.4)	86 (28.8)	21 (7.0)	71 (23.7)	58 (19.4)
Palpate the abdomen for tenderness and distension	27 (9.00)	76 (25.4)	14 (4.7)	46 (15.4)	78 (26.1)	58 (19.4)
Palpate extremities for tenderness	25 (8.4)	68 (22.7)	28 (9.4)	42 (14.0)	85 (28.4)	51 (17.1)
Assess muscle strength	18 (6.0)	43 (14.4)	39 (13.0)	88 (29.4)	38 (12.7)	73 (24.4)
Evaluate speech pattern	51 (17.0)	43 (14.4)	32 (10.7)	71 (23.7)	37 (12.4)	65 (21.7)
Assess hearing based on a conversation	37 (12.4)	58 (19.4)	28 (9.4)	90 (30.1)	29 (9.7)	57 (19.1)
Measure BP using a sphygmomanometer	22 (7.4)	15 (5.0)	39 (13.0)	28 (9.4)	51 (17.1)	144 (48.2)
Measure body temperature	7 (2.3)	33 (11.0)	29 (9.7)	21 (7.0)	57 (19.1)	152 (50.8)
Measure oxygen saturation using pulse-oximeters	7 (2.3)	16 (5.4)	11 (3.7)	49 (16.4)	89 (29.8)	127 (42.5)
NGT and patient monitoring machines	7 (2.3)	26 (8.7)	22 (7.4)	55 (18.4)	72 (24.1)	117 (39.1)

Note: GCS: Glasgow Coma Scale; BP: blood pressure; NGT: nasogastric tube.

**Table 3:** Barriers towards physical assessment among nurses working in ICU at Amhara regional state referral hospitals, Northwest Ethiopia, 2019 (*n* = 299).

Physical assessment barriers items	M (SD)	Agree *n* (%)	Disagree *n* (%)
Subscale 1: reliance on others and technology 29.27 (±7.48)			
It is not the nurse's role to conduct a physical assessment of the patient	2.27 (3.81)	95 (31.8)	204 (68.2)
Gather all physical assessment data using electronic monitoring devices	2.66 (1.43)	130 (43.5)	169 (56.5)
Use of technology reduces the need for nurses' physical assessment skills	2.31 (1.33)	113 (37.8)	186 (62.2)
Nurses do not need to use many physical assessment skills to do their job well	2.22 (1.29)	82 (27.4)	217 (72.6)
Physical assessment is something only the doctor does	2.01 (1.32)	70 (23.4)	228 (76.3)
Relying on monitoring equipment to collect assessment data	3.15 (1.41)	168 (56.2)	131 (43.8)
Physical assessment is used only when a patient deteriorates	1.66 (1.180	100 (33.4)	198 (66.2)
Physical assessment is the responsibility of medical or allied health staff	3.27 (1.49)	176 (58.9)	123 (41.1)
Do not use physical assessment skills because of the task-oriented nature of the work	2.59 (1.48)	124 (41.5)	175 (58.5)
Subscale 2: lack of time and interruptions 12.21 (±4.31)			
Lack of time is a barrier in using physical assessment skills	2.60 (1.20)	139 (46.5)	160 (53.5)
Lack of time to do in-depth physical assessment to the patients	2.95 (1.275)	181 (60.5)	118 (39.5)
No time to use physical assessment skills because of the workload	2.87 (1.33)	147 (49.2)	152 (50.8)
Completing checklists and documentation means no time to use physical assessment skills	2.90 (1.33)	155 (51.8)	144 (48.2)
Too many interruptions during work prevent from doing a physical assessment	2.79 (1.52)	146 (48.8)	153 (51.2)
Subscale 3: ward culture 13.84 (±6.07)			
The ward culture is a barrier in using physical assessment skills	2.80 (1.230	169 (56.5)	130 (43.5)
Assessment is done a certain way in the ward which limits the extent of physical assessment	3.11 (1.42)	170 (56.9)	129 (43.1)
Assessments I make using physical assessment skills are not valued by my coworkers	2.75 (1.52)	129 (43.1)	170 (56.9)
The ward culture discourages nurses from doing a physical assessment in my workplace	2.81 (1.520	143 (47.8)	156 (52.2)
Feel of support by colleagues to use physical assessment skills	3.28 (3.35)	154 (51.5)	145 (48.5)
Subscale 4: lack of confidence 12.18 (±4.18)			
Lack of confidence in accurately performing physical assessment	2.82 (1.39)	147 (49.2)	152 (50.8)
Worrying about the ability to correctly use physical assessment skills	3.00 (1.468)	150 (50.2)	149 (49.8)
Lack of confidence in deciding what physical assessment skills to use	3.08 (1.57)	150 (50.2)	149 (49.8)
Competently use physical assessment skills	3.69 (1.35)	213 (71.2)	86 (28.8)
Subscale 5: lack of nursing role models 12.36 (±4.13)			
Physical assessment skills are a role model by experienced nurses in the ward	3.08 (1.540	174 (58.2)	125 (41.8)
Nurse leaders promote the use of physical assessment skills in the unit classroom	3.14 (1.53)	159 (53.2)	140 (46.8)
Nurses encourage each other to use physical assessment skills in the ward	3.44 (1.25)	201 (67.2)	98 (32.8)
There is a lack of experienced nursing staff to role model physical assessment skills in the	3.24 (1.54)	182 (60.9)	117 (39.1)
Subscale 6: lack of influence on patient care 12.68 (±5.28)			
Information on physical assessment skills is used to develop a plan of care	3.33 (1.390	194 (64.9)	105 (35.1)
The ability to use physical assessment skills makes a positive difference in patient care	3.58 (1.720	220 (73.6)	79 (26.4)
The ability to use physical assessment skills improves the quality of nursing care	3.69 (1.30)	206 (68.9)	93 (31.1)
The information collected using physical assessment skills is used to make treatment decisions	3.86 (3.28)	217 (72.6)	82 (27.4)
Subscale 7: specialty area 13.02 (±4.37)			
Physical assessment skills are relevant to nurses in the specialty area	3.24 (1.58)	185 (61.9)	114 (38.1)
Do not use physical assessment skills that are outside of the specialty area	3.04 (1.43)	162 (54.2)	137 (45.8)
The specialty area determines the physical assessment skills that nurses used	2.98 (1.23)	190 (63.5)	109 (36.5)
Physical assessment skills are restricted only to specialty area	2.64 (1.09)	186 (62.2)	113 (37.8)
Physical assessment skills determined by what is acceptable on the ward	3.31 (0.96)	129 (43.1)	170 (56.9)

Note: M: mean; SD: standard deviation.

**Table 4:** Factors associated with the practice of nurses working in ICU towards physical assessment at Amhara regional state referral hospitals, Northwest Ethiopia, 2019 (*n* = 299).

Variables	Categories	Crude unstandardized *β* coefficient (95% CI)	Adjusted unstandardized *β* coefficient (95% CI)
Sex	Male	0	0
	Female	6.79 (1.13, 12.46)∗	3.89 (-0.58, 8.35)
Marital status	Married	0	0
	Unmarried	0.83 (-5.29, 6.94)	6.10 (1.73, 10.46)∗∗
Educational level	Diploma	0	0
	Degree	-1.86 (-19.56, 15.87)	9.84 (-3.62, 23.31)
	Master	0.32 (-18.70, 19.35)	10.58 (-4.41, 25.57)
Training	No	0	0
	Yes	22.49 (17.22, 27.75)∗	11.53 (6.34, 16.72)∗∗
Age in years		0.94 (0.20, 1.68)∗	-0.451 (-1.273, 0.372)
Total years of experience as a nurse		1.97 (0.87, 3.07)∗	1.368 (-0.234, 2.971)
Years of experience working in ICU		4.70 (1.17, 8.24)∗	-0.289 (-3.208, 2.629)
Knowledge		4.74 (3.96, 5.52)∗	2.81 (2.00, 3.63)∗∗
Attitude		1.81 (1.40, 2.22)∗	0.21 (-0.20, 0.62)
Reliance on others and technology		-1.48 (-1.83, -1.14)∗	-0.78 (-1.07, -0.48)∗∗
Lack of time and interruption		-1.67 (-2.30, -1.03)∗	0.160 (-0.500, 0.821)
Ward culture		-1.215 (-1.665, -0.766)∗	-0.48 (-0.85, -0.11)∗∗
Lack of confidence		-1.328 (-1.993, -0.663)∗	-0.16 (-0.71, 0.40)
Lack of nursing role model		-0.685 (-1.371, -0.001)∗	-0.54 (-1.06, -0.02)∗∗
Lack of influence on patient care		-0.417 (-0.956, 0.121)	0.191 (-0.21, 0.59)
Specialty area		-2.88 (-3.44, -2.31)∗	-1.46 (-2.01, -0.90)∗∗

Note: ∗ significant at *p* < 0.05 (crude unstandardized *β* coefficient (95% CI)), ∗∗ significant at *p* value < 0.05 (adjusted unstandardized *β* coefficient (95% CI)), CI: confidence interval; ICU: intensive care units.

## Data Availability

All data about this study are contained and presented in this document.

## Abbreviations

CI: Confidence interval
FMOH: Federal ministry of health
ICUs: Intensive care units
SD: Standard deviation.
